# Crystal structure of oryzalin

**DOI:** 10.1107/S205698901500955X

**Published:** 2015-05-30

**Authors:** Gihaeng Kang, Jineun Kim, Youngeun Jeon, Tae Ho Kim

**Affiliations:** a Department of Chemistry and Research Institute of Natural Sciences, Gyeongsang National University, Jinju 660-701, Republic of Korea

**Keywords:** crystal structure, oryzalin, sulfonamide, herbicidal properties, hydrogen bonding

## Abstract

The title compound, C_12_H_18_N_4_O_6_S (systematic name: 4-di­propyl­amino-3,5-di­nitro­benzene­sulfonamide), is a sulfonamide with herbicidal properties marketed as oryzalin. The dihedral angles between the benzene ring and the mean planes of the nitro groups are 26.15 (11) and 54.80 (9)°. The propyl arms of the di­propyl­amino substituent lie on opposite sides of this ring plane. In the crystal, N—H⋯O and C—H⋯O hydrogen bonds generate a three-dimensional network.

## Related literature   

For information on the toxicity and herbicidal properties of the title compound, see: Naqvi & Leung (1983 ▸). For related crystal structures, see: O’Connell & Maslen (1967 ▸); Tremayne *et al.* (2002 ▸).
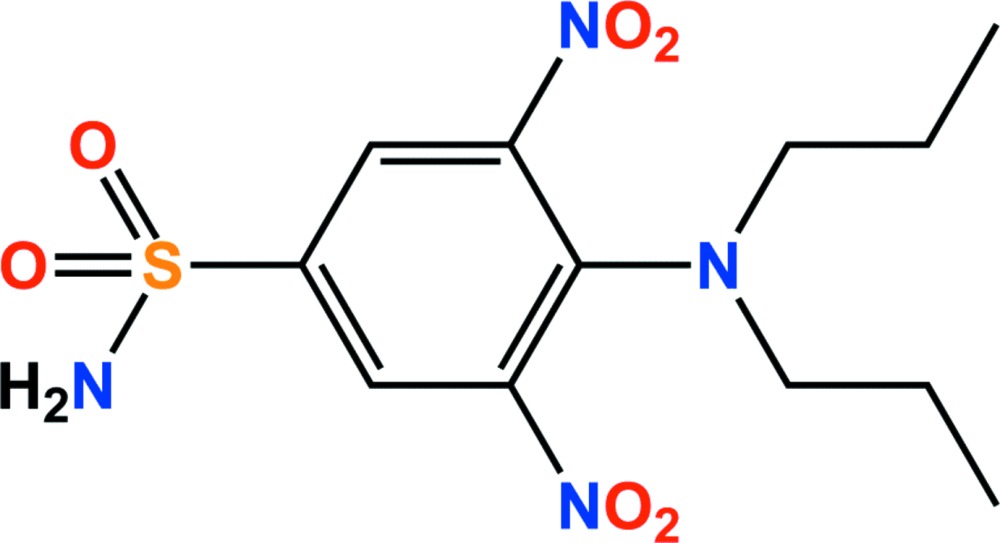


## Experimental   

### Crystal data  

C_12_H_18_N_4_O_6_S
*M* *_r_* = 346.36
Triclinic, 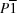

*a* = 7.6057 (2) Å
*b* = 8.2463 (2) Å
*c* = 12.8657 (2) Å
α = 73.901 (1)°
β = 86.059 (1)°
γ = 83.549 (1)°
*V* = 769.77 (3) Å^3^
*Z* = 2
Mo *K*α radiation
μ = 0.25 mm^−1^
*T* = 173 K
0.49 × 0.17 × 0.05 mm

### Data collection  

Bruker APEXII CCD diffractometer
Absorption correction: multi-scan (*SADABS*; Bruker, 2009 ▸) *T*_min_ = 0.888, *T*_max_ = 0.988
14199 measured reflections
3778 independent reflections
3506 reflections with *I* > 2σ(*I*)
*R*_int_ = 0.021

### Refinement  

*R*[*F* ^2^ > 2σ(*F* ^2^)] = 0.033
*wR*(*F* ^2^) = 0.090
*S* = 1.05
3778 reflections
218 parameters
H atoms treated by a mixture of independent and constrained refinement
Δρ_max_ = 0.29 e Å^−3^
Δρ_min_ = −0.42 e Å^−3^

### 

Data collection: *APEX2* (Bruker, 2009 ▸); cell refinement: *SAINT* (Bruker, 2009 ▸); data reduction: *SAINT*; program(s) used to solve structure: *SHELXTL* (Sheldrick, 2008 ▸); program(s) used to refine structure: *SHELXTL*; molecular graphics: *DIAMOND* (Brandenburg, 2010 ▸); software used to prepare material for publication: *SHELXTL*.

## Supplementary Material

Crystal structure: contains datablock(s) global, I. DOI: 10.1107/S205698901500955X/sj5462sup1.cif

Structure factors: contains datablock(s) I. DOI: 10.1107/S205698901500955X/sj5462Isup2.hkl

Supporting information file. DOI: 10.1107/S205698901500955X/sj5462Isup3.cml

**Fig. 1.**
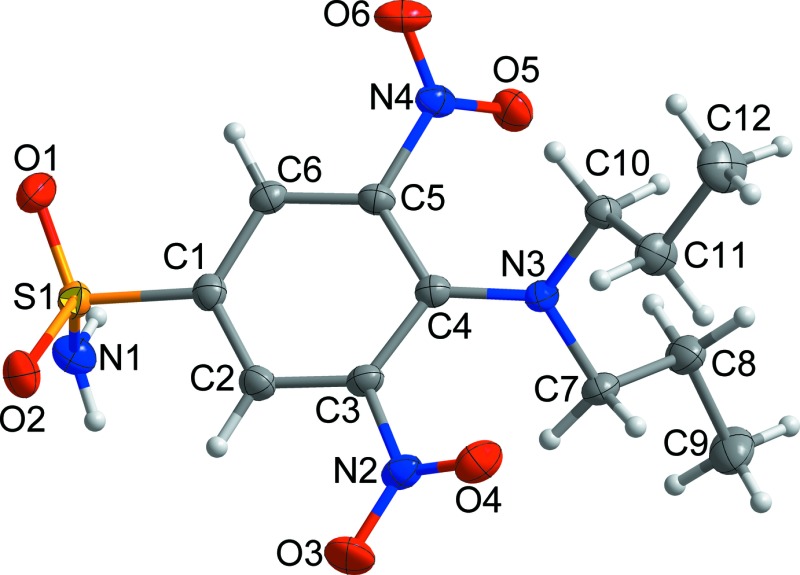
The asymmetric unit of the title compound with the atom numbering scheme. Displacement ellipsoids are drawn at the 50% probability level. H atoms are shown as small spheres of arbitrary radius.

**Fig. 2.**
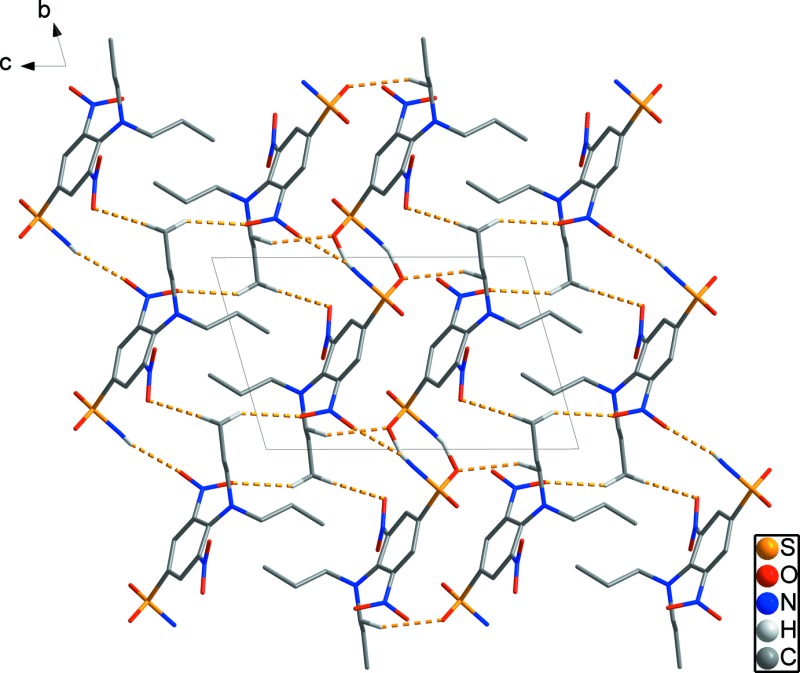
Crystal packing viewed along the a axis. The intermolecular N—H···O and C—H···O hydrogen bonds are shown as dashed lines.

. DOI: 10.1107/S205698901500955X/sj5462fig1.tif
The asymmetric unit of the title compound with the atom numbering scheme. Displacement ellipsoids are drawn at the 50% probability level. H atoms are shown as small spheres of arbitrary radius.

a . DOI: 10.1107/S205698901500955X/sj5462fig2.tif
Crystal packing viewed along the *a* axis. The inter­molecular N—H⋯O and C—H⋯O hydrogen bonds are shown as dashed lines.

CCDC reference: 1401628

Additional supporting information:  crystallographic information; 3D view; checkCIF report

## Figures and Tables

**Table 1:** Hydrogen-bond geometry (, )

*D*H*A*	*D*H	H*A*	*D* *A*	*D*H*A*
N1H2*N*O6^i^	0.86(2)	2.529(19)	2.9956(15)	114.9(15)
N1H2*N*O2^ii^	0.86(2)	2.26(2)	3.0839(16)	160.5(17)
N1H1*N*O3^iii^	0.81(2)	2.15(2)	2.9474(16)	170.0(19)
C2H2N1^ii^	0.95	2.74	3.6843(17)	171
C9H9*A*O4^iv^	0.98	2.69	3.3192(18)	122
C10H10*A*O2^v^	0.99	2.59	3.4033(15)	140
C12H12*C*O3^vi^	0.98	2.71	3.249(2)	115
C12H12*A*O5^vii^	0.98	2.61	3.492(2)	150

Symmetry codes: (i) 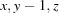; (ii) 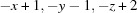; (iii) 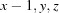; (iv) 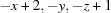; (v) 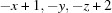; (vi) 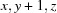; (vii) 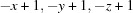.

## supplementary crystallographic information

### S1. Comment

Oryzalin, C_12_H_18_N_4_O_6_S, is a sulfonamide herbicide for soybean and crop weeds (Naqvi & Leung, 1983). Its crystal structure is reported herein. In this compound (Scheme 1, Fig. 1), the dihedral angles between the central phenyl ring and the mean planes of two nitro groups are 26.15 (11) and 54.80 (9)°, respectively. All bond lengths and bond angles are normal and comparable to those observed in the crystal structures of similar compounds (O'Connell & Maslen, 1967; Tremayne *et al.*, 2002).

In the crystal structure (Fig. 2), the crystal structure is stabilized by intermolecular N—H···O and C—H···O hydrogen bonds (Table 1), resulting in a three-dimensional architechture.

### S2. Experimental

The title compound was purchased from the Dr. Ehrenstorfer GmbH Company. Slow evaporation of a solution in CH_2_Cl_2_ gave single crystals suitable for X-ray analysis.

### S3. Refinement

The N-bound H atoms were located in a difference Fourier map and freely refined (N—H = 0.81 (2) - 0.86 (2) Å). The C-bound H atoms were positioned geometrically and refined using a riding model with d(C—H) = 0.98 Å, *U*_iso_ = 1.2*U*_eq_(C) for methyl group, d(C—H) = 0.99 Å, *U*_iso_ = 1.2*U*_eq_(C) for C*sp*^3^—H, and d(C—H) = 0.95 Å, *U*_iso_ = 1.2*U*_eq_(C) for aromatic C—H.

### Crystal data

**Table:**

C_12_H_18_N_4_O_6_S	*Z* = 2
*M**_r_* = 346.36	*F*(000) = 364
Triclinic, *P*1	*D*_x_ = 1.494 Mg m^−^^3^
*a* = 7.6057 (2) Å	Mo *K*α radiation, λ = 0.71073 Å
*b* = 8.2463 (2) Å	Cell parameters from 9733 reflections
*c* = 12.8657 (2) Å	θ = 2.6–28.3°
α = 73.901 (1)°	µ = 0.25 mm^−^^1^
β = 86.059 (1)°	*T* = 173 K
γ = 83.549 (1)°	Block, red
*V* = 769.77 (3) Å^3^	0.49 × 0.17 × 0.05 mm

### Data collection

**Table:**

Bruker APEXII CCD diffractometer	3778 independent reflections
Radiation source: fine-focus sealed tube	3506 reflections with *I* > 2σ(*I*)
Graphite monochromator	*R*_int_ = 0.021
φ and ω scans	θ_max_ = 28.3°, θ_min_ = 2.6°
Absorption correction: multi-scan (*SADABS*; Bruker, 2009)	*h* = −10→8
*T*_min_ = 0.888, *T*_max_ = 0.988	*k* = −10→10
14199 measured reflections	*l* = −17→17

### Refinement

**Table:**

Refinement on *F*^2^	Primary atom site location: structure-invariant direct methods
Least-squares matrix: full	Secondary atom site location: difference Fourier map
*R*[*F*^2^ > 2σ(*F*^2^)] = 0.033	Hydrogen site location: inferred from neighbouring sites
*wR*(*F*^2^) = 0.090	H atoms treated by a mixture of independent and constrained refinement
*S* = 1.05	*w* = 1/[σ^2^(*F*_o_^2^) + (0.0441*P*)^2^ + 0.3414*P*] where *P* = (*F*_o_^2^ + 2*F*_c_^2^)/3
3778 reflections	(Δ/σ)_max_ = 0.002
218 parameters	Δρ_max_ = 0.29 e Å^−^^3^
0 restraints	Δρ_min_ = −0.42 e Å^−^^3^

### Special details

**Table:**

Geometry. All e.s.d.'s (except the e.s.d. in the dihedral angle between two l.s. planes) are estimated using the full covariance matrix. The cell e.s.d.'s are taken into account individually in the estimation of e.s.d.'s in distances, angles and torsion angles; correlations between e.s.d.'s in cell parameters are only used when they are defined by crystal symmetry. An approximate (isotropic) treatment of cell e.s.d.'s is used for estimating e.s.d.'s involving l.s. planes.
Refinement. Refinement of *F*^2^ against ALL reflections. The weighted *R*-factor *wR* and goodness of fit *S* are based on *F*^2^, conventional *R*-factors *R* are based on *F*, with *F* set to zero for negative *F*^2^. The threshold expression of *F*^2^ > σ(*F*^2^) is used only for calculating *R*-factors(gt) *etc*. and is not relevant to the choice of reflections for refinement. *R*-factors based on *F*^2^ are statistically about twice as large as those based on *F*, and *R*- factors based on ALL data will be even larger.

### Fractional atomic coordinates and isotropic or equivalent isotropic displacement parameters (Å^2^)

**Table:**

	*x*	*y*	*z*	*U*_iso_*/*U*_eq_	
S1	0.29089 (4)	−0.28775 (4)	1.01726 (2)	0.02217 (9)	
O6	0.21891 (13)	0.38350 (13)	0.79517 (9)	0.0345 (2)	
O5	0.27982 (13)	0.32749 (13)	0.64100 (8)	0.0310 (2)	
O4	0.94829 (12)	0.02295 (14)	0.76530 (8)	0.0328 (2)	
O3	0.91096 (14)	−0.24191 (14)	0.83339 (10)	0.0413 (3)	
O2	0.42149 (13)	−0.38814 (12)	1.09026 (7)	0.0293 (2)	
O1	0.13725 (14)	−0.20406 (13)	1.05726 (8)	0.0337 (2)	
N1	0.22714 (17)	−0.41143 (15)	0.95364 (10)	0.0268 (2)	
N3	0.65122 (14)	0.20712 (13)	0.66209 (8)	0.0207 (2)	
N4	0.29355 (13)	0.29708 (13)	0.73912 (9)	0.0221 (2)	
N2	0.85394 (14)	−0.09134 (15)	0.80100 (8)	0.0252 (2)	
C1	0.40261 (16)	−0.13002 (15)	0.92262 (9)	0.0209 (2)	
C6	0.31660 (16)	0.03024 (15)	0.87675 (9)	0.0201 (2)	
H6	0.2012	0.0621	0.9021	0.024*	
C5	0.40260 (15)	0.14133 (14)	0.79389 (9)	0.0189 (2)	
C4	0.57601 (15)	0.10563 (14)	0.75238 (9)	0.0184 (2)	
C10	0.64961 (16)	0.39061 (15)	0.64526 (10)	0.0210 (2)	
H10A	0.5757	0.4247	0.7035	0.025*	
H10B	0.5958	0.4507	0.5753	0.025*	
C11	0.83533 (18)	0.44270 (17)	0.64495 (12)	0.0298 (3)	
H11A	0.8881	0.3855	0.7156	0.036*	
H11B	0.9104	0.4062	0.5880	0.036*	
C12	0.8312 (2)	0.63353 (19)	0.62429 (14)	0.0386 (3)	
H12A	0.7900	0.6898	0.5514	0.058*	
H12B	0.9506	0.6633	0.6304	0.058*	
H12C	0.7506	0.6707	0.6778	0.058*	
C7	0.74354 (17)	0.13316 (16)	0.57981 (10)	0.0235 (2)	
H7A	0.7354	0.0092	0.6013	0.028*	
H7B	0.8704	0.1524	0.5751	0.028*	
C8	0.66410 (19)	0.21110 (18)	0.46926 (10)	0.0298 (3)	
H8A	0.5335	0.2132	0.4772	0.036*	
H8B	0.6939	0.3296	0.4412	0.036*	
C9	0.7322 (2)	0.1124 (2)	0.38841 (12)	0.0384 (3)	
H9A	0.8618	0.1057	0.3828	0.058*	
H9B	0.6843	0.1703	0.3174	0.058*	
H9C	0.6943	−0.0023	0.4130	0.058*	
C3	0.66129 (15)	−0.05195 (15)	0.81163 (9)	0.0207 (2)	
C2	0.57619 (16)	−0.16972 (16)	0.89134 (10)	0.0227 (2)	
H2	0.6365	−0.2771	0.9244	0.027*	
H2N	0.312 (3)	−0.469 (2)	0.9280 (15)	0.040 (5)*	
H1N	0.145 (3)	−0.371 (3)	0.9149 (16)	0.044 (5)*	

### Atomic displacement parameters (Å^2^)

**Table:**

	*U*^11^	*U*^22^	*U*^33^	*U*^12^	*U*^13^	*U*^23^
S1	0.02480 (16)	0.02270 (15)	0.01851 (14)	−0.00303 (11)	0.00036 (11)	−0.00479 (11)
O6	0.0289 (5)	0.0317 (5)	0.0472 (6)	0.0089 (4)	−0.0057 (4)	−0.0212 (5)
O5	0.0278 (5)	0.0336 (5)	0.0268 (5)	0.0022 (4)	−0.0057 (4)	−0.0013 (4)
O4	0.0195 (4)	0.0433 (6)	0.0375 (5)	−0.0035 (4)	0.0010 (4)	−0.0147 (4)
O3	0.0284 (5)	0.0378 (6)	0.0472 (6)	0.0141 (4)	−0.0041 (5)	−0.0002 (5)
O2	0.0337 (5)	0.0318 (5)	0.0202 (4)	−0.0041 (4)	−0.0062 (4)	−0.0021 (4)
O1	0.0338 (5)	0.0322 (5)	0.0332 (5)	−0.0025 (4)	0.0117 (4)	−0.0093 (4)
N1	0.0269 (6)	0.0247 (5)	0.0297 (6)	−0.0011 (5)	−0.0079 (5)	−0.0078 (4)
N3	0.0221 (5)	0.0188 (5)	0.0212 (5)	−0.0008 (4)	0.0033 (4)	−0.0067 (4)
N4	0.0164 (5)	0.0198 (5)	0.0300 (5)	−0.0005 (4)	−0.0028 (4)	−0.0065 (4)
N2	0.0197 (5)	0.0344 (6)	0.0208 (5)	0.0053 (4)	−0.0031 (4)	−0.0087 (4)
C1	0.0230 (6)	0.0219 (5)	0.0182 (5)	−0.0033 (4)	−0.0005 (4)	−0.0058 (4)
C6	0.0186 (5)	0.0225 (5)	0.0213 (5)	−0.0017 (4)	−0.0008 (4)	−0.0095 (4)
C5	0.0177 (5)	0.0185 (5)	0.0213 (5)	0.0006 (4)	−0.0036 (4)	−0.0068 (4)
C4	0.0181 (5)	0.0195 (5)	0.0190 (5)	−0.0011 (4)	−0.0022 (4)	−0.0075 (4)
C10	0.0220 (6)	0.0183 (5)	0.0232 (5)	−0.0013 (4)	−0.0008 (4)	−0.0069 (4)
C11	0.0233 (6)	0.0284 (6)	0.0372 (7)	−0.0062 (5)	−0.0001 (5)	−0.0069 (5)
C12	0.0386 (8)	0.0320 (7)	0.0487 (9)	−0.0143 (6)	−0.0012 (7)	−0.0127 (6)
C7	0.0244 (6)	0.0233 (6)	0.0229 (6)	0.0007 (5)	0.0038 (4)	−0.0090 (5)
C8	0.0343 (7)	0.0312 (7)	0.0231 (6)	0.0029 (6)	0.0011 (5)	−0.0088 (5)
C9	0.0531 (9)	0.0376 (8)	0.0278 (7)	−0.0050 (7)	0.0051 (6)	−0.0155 (6)
C3	0.0169 (5)	0.0247 (6)	0.0204 (5)	0.0023 (4)	−0.0015 (4)	−0.0077 (4)
C2	0.0238 (6)	0.0221 (5)	0.0207 (5)	0.0023 (5)	−0.0034 (4)	−0.0046 (4)

### Geometric parameters (Å, º)

**Table:**

S1—O1	1.4282 (10)	C10—C11	1.5213 (17)
S1—O2	1.4375 (10)	C10—H10A	0.9900
S1—N1	1.6043 (12)	C10—H10B	0.9900
S1—C1	1.7675 (12)	C11—C12	1.519 (2)
O6—N4	1.2199 (14)	C11—H11A	0.9900
O5—N4	1.2264 (14)	C11—H11B	0.9900
O4—N2	1.2168 (16)	C12—H12A	0.9800
O3—N2	1.2330 (15)	C12—H12B	0.9800
N1—H2N	0.86 (2)	C12—H12C	0.9800
N1—H1N	0.81 (2)	C7—C8	1.5248 (18)
N3—C4	1.3617 (15)	C7—H7A	0.9900
N3—C7	1.4648 (14)	C7—H7B	0.9900
N3—C10	1.4666 (15)	C8—C9	1.5208 (19)
N4—C5	1.4741 (15)	C8—H8A	0.9900
N2—C3	1.4701 (15)	C8—H8B	0.9900
C1—C2	1.3838 (17)	C9—H9A	0.9800
C1—C6	1.3961 (17)	C9—H9B	0.9800
C6—C5	1.3786 (17)	C9—H9C	0.9800
C6—H6	0.9500	C3—C2	1.3826 (17)
C5—C4	1.4193 (16)	C2—H2	0.9500
C4—C3	1.4211 (16)		
			
O1—S1—O2	120.49 (6)	C12—C11—C10	110.85 (11)
O1—S1—N1	108.20 (7)	C12—C11—H11A	109.5
O2—S1—N1	106.10 (6)	C10—C11—H11A	109.5
O1—S1—C1	107.20 (6)	C12—C11—H11B	109.5
O2—S1—C1	106.46 (6)	C10—C11—H11B	109.5
N1—S1—C1	107.83 (6)	H11A—C11—H11B	108.1
S1—N1—H2N	114.6 (13)	C11—C12—H12A	109.5
S1—N1—H1N	114.7 (14)	C11—C12—H12B	109.5
H2N—N1—H1N	116.7 (19)	H12A—C12—H12B	109.5
C4—N3—C7	120.12 (10)	C11—C12—H12C	109.5
C4—N3—C10	122.12 (10)	H12A—C12—H12C	109.5
C7—N3—C10	117.72 (10)	H12B—C12—H12C	109.5
O6—N4—O5	124.71 (11)	N3—C7—C8	111.23 (10)
O6—N4—C5	117.62 (10)	N3—C7—H7A	109.4
O5—N4—C5	117.63 (10)	C8—C7—H7A	109.4
O4—N2—O3	123.62 (11)	N3—C7—H7B	109.4
O4—N2—C3	119.83 (11)	C8—C7—H7B	109.4
O3—N2—C3	116.50 (11)	H7A—C7—H7B	108.0
C2—C1—C6	120.07 (11)	C9—C8—C7	111.89 (12)
C2—C1—S1	118.82 (9)	C9—C8—H8A	109.2
C6—C1—S1	121.07 (9)	C7—C8—H8A	109.2
C5—C6—C1	118.64 (11)	C9—C8—H8B	109.2
C5—C6—H6	120.7	C7—C8—H8B	109.2
C1—C6—H6	120.7	H8A—C8—H8B	107.9
C6—C5—C4	124.46 (11)	C8—C9—H9A	109.5
C6—C5—N4	115.18 (10)	C8—C9—H9B	109.5
C4—C5—N4	119.93 (10)	H9A—C9—H9B	109.5
N3—C4—C5	123.91 (10)	C8—C9—H9C	109.5
N3—C4—C3	122.89 (10)	H9A—C9—H9C	109.5
C5—C4—C3	113.13 (10)	H9B—C9—H9C	109.5
N3—C10—C11	111.69 (10)	C2—C3—C4	123.50 (11)
N3—C10—H10A	109.3	C2—C3—N2	114.89 (10)
C11—C10—H10A	109.3	C4—C3—N2	121.19 (11)
N3—C10—H10B	109.3	C3—C2—C1	119.54 (11)
C11—C10—H10B	109.3	C3—C2—H2	120.2
H10A—C10—H10B	107.9	C1—C2—H2	120.2
			
O1—S1—C1—C2	164.43 (10)	C6—C5—C4—C3	−5.59 (16)
O2—S1—C1—C2	34.23 (11)	N4—C5—C4—C3	−177.72 (10)
N1—S1—C1—C2	−79.27 (11)	C4—N3—C10—C11	114.40 (13)
O1—S1—C1—C6	−17.88 (12)	C7—N3—C10—C11	−63.22 (14)
O2—S1—C1—C6	−148.07 (10)	N3—C10—C11—C12	178.41 (11)
N1—S1—C1—C6	98.42 (11)	C4—N3—C7—C8	122.80 (12)
C2—C1—C6—C5	5.48 (17)	C10—N3—C7—C8	−59.53 (14)
S1—C1—C6—C5	−172.19 (9)	N3—C7—C8—C9	−168.42 (12)
C1—C6—C5—C4	−1.23 (17)	N3—C4—C3—C2	−168.24 (11)
C1—C6—C5—N4	171.23 (10)	C5—C4—C3—C2	8.86 (17)
O6—N4—C5—C6	54.74 (15)	N3—C4—C3—N2	19.50 (17)
O5—N4—C5—C6	−122.95 (12)	C5—C4—C3—N2	−163.40 (10)
O6—N4—C5—C4	−132.43 (12)	O4—N2—C3—C2	−153.30 (11)
O5—N4—C5—C4	49.88 (15)	O3—N2—C3—C2	24.18 (16)
C7—N3—C4—C5	−133.66 (12)	O4—N2—C3—C4	19.59 (17)
C10—N3—C4—C5	48.78 (16)	O3—N2—C3—C4	−162.93 (12)
C7—N3—C4—C3	43.12 (16)	C4—C3—C2—C1	−5.24 (18)
C10—N3—C4—C3	−134.44 (12)	N2—C3—C2—C1	167.46 (11)
C6—C5—C4—N3	171.47 (11)	C6—C1—C2—C3	−2.40 (18)
N4—C5—C4—N3	−0.66 (17)	S1—C1—C2—C3	175.32 (9)

### Hydrogen-bond geometry (Å, º)

**Table:**

*D*—H···*A*	*D*—H	H···*A*	*D*···*A*	*D*—H···*A*
N1—H2*N*···O6^i^	0.86 (2)	2.529 (19)	2.9956 (15)	114.9 (15)
N1—H2*N*···O2^ii^	0.86 (2)	2.26 (2)	3.0839 (16)	160.5 (17)
N1—H1*N*···O3^iii^	0.81 (2)	2.15 (2)	2.9474 (16)	170.0 (19)
C2—H2···N1^ii^	0.95	2.74	3.6843 (17)	171
C9—H9*A*···O4^iv^	0.98	2.69	3.3192 (18)	122
C10—H10*A*···O2^v^	0.99	2.59	3.4033 (15)	140
C12—H12*C*···O3^vi^	0.98	2.71	3.249 (2)	115
C12—H12*A*···O5^vii^	0.98	2.61	3.492 (2)	150

Symmetry codes: (i) *x*, *y*−1, *z*; (ii) −*x*+1, −*y*−1, −*z*+2; (iii) *x*−1, *y*, *z*; (iv) −*x*+2, −*y*, −*z*+1; (v) −*x*+1, −*y*, −*z*+2; (vi) *x*, *y*+1, *z*; (vii) −*x*+1, −*y*+1, −*z*+1.
