# Supplementary figures and images for: Crystal structure of oryzalin
Source: Acta Crystallogr E Crystallogr Commun. 2015 May 30;71(Pt 6):o429. doi: 10.1107/S205698901500955X (PMC4459385; doi:10.1107/S205698901500955X)

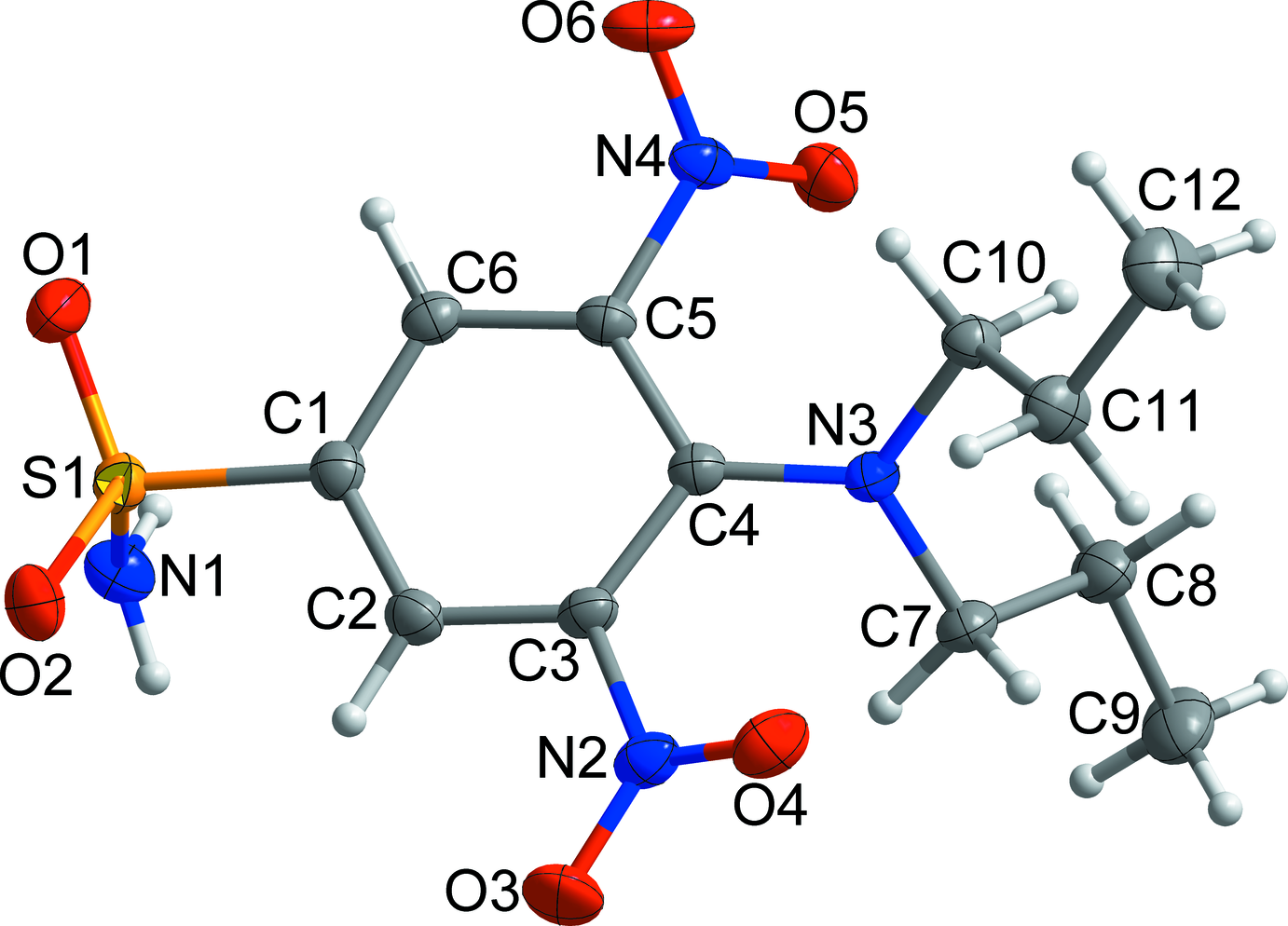

Supplement: Supplementary file 4 [file e-71-0o429-fig1.tif]

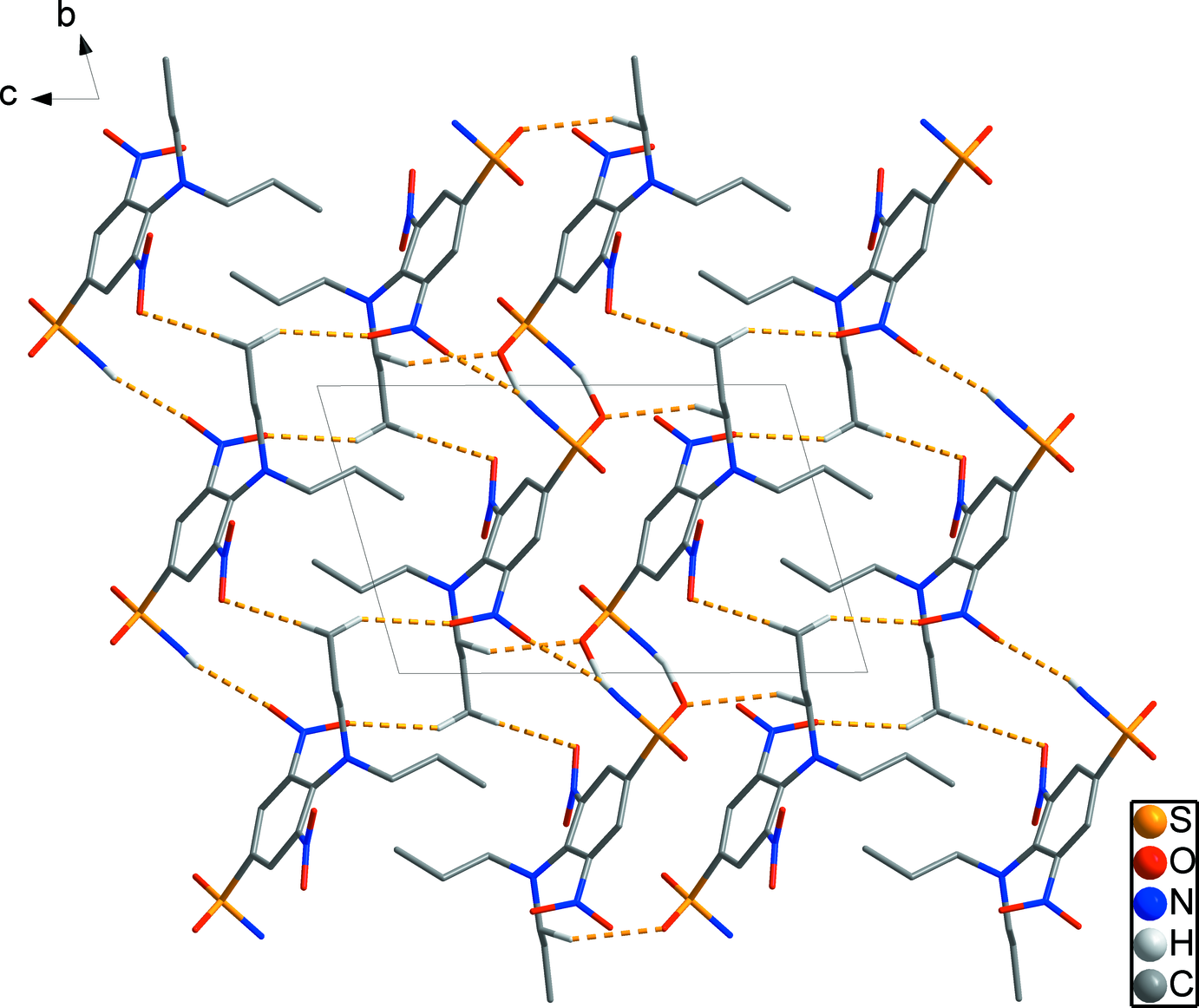

Supplement: Supplementary file 5 [file e-71-0o429-fig2.tif]
